# LC–MS/MS separation and quantitation of ribavirin in chicken and comparison of different mass spectrometric platforms

**DOI:** 10.1186/s13065-023-01010-4

**Published:** 2023-08-07

**Authors:** Daokun Xu, Haolun Huang, Wenyan Hu, Xinmei Liu, Jun Yang

**Affiliations:** 1 Nanjing Institute for Food and Drug Control, Nanjing, 211198 Jiangsu Province People’s Republic of China; 2 State Key Laboratory of Food Science and Technology, School of Food Science and Technology, Jiangnan University, Wuxi, 214122 Jiangsu Province People’s Republic of China; 3 Demonstration Collaborative Laboratory of Analysis and Detection Technology for Food and Drug Safety, Nanjing Institute for Food and Drug Control and Agilent Technologies (China), Nanjing, 211198 Jiangsu Province People’s Republic of China; 4 Collaborative Laboratory for Food Safety, Nanjing Institute for Food and Drug Control and SCIEX (China), Nanjing, 211198 Jiangsu Province People’s Republic of China

**Keywords:** LC–MS/MS, Anti–viral drug, Ribavirin, Separation, Chicken

## Abstract

A liquid chromatography–tandem mass spectrometry (LC–MS/MS) method was developed for the analysis of ribavirin in chicken. Samples was extracted with 0.1% formic acid and purified by Hypercarb cartridge prior to LC–MS/MS analysis. The eluates were evaporated to dryness, reconstituted in 1 mL 5mM ammonium acetate containing 5% acetonitrile (v/v) and 0.1% (v/v) formic acid. Chromatographic separation was performed on a Hypercarb analytical column under a gradient elution program with acetonitrile and 0.1% (v/v) formic acid in 5 mM ammonium acetate at a flow rate of 0.6 ml/min. The intraday and interday accuracy ranged from − 7.83 − 1.39%, and − 6.38 − 2.25%, with precisions between 1.34 − 3.88%% and 1.10 − 4.67%. The limits of detection (LODs) and limits of quantitation (LOQs) of ribavirin was 0.1 ng/mL and 0.5 ng/mL, respectively. The method was validated for linearity, accuracy, precision, matrix effect and stability. Application of the method confirmed 3 ribavirin positive samples out of 50 commercial chicken samples, with concentrations of ribavirin ranging from 0.9 μg/kg to 5.8 μg/kg a, respectively. Additionally, both AB Sciex 5500 and Agilent 6945B were proven to be suitable in ribavirin separation and quantification. The described method is suitable for the determination of ribavirin in chicken in analytical practice to monitor illegal addition of this kind of anti–viral drug.

**Supplementary Information:**

The online version contains supplementary material available at 10.1186/s13065-023-01010-4.

## Introduction

Ribavirin is a nucleoside analogue with a broad–spectrum antivirus activity. It has been used extensively in the treatment of respiratory syncytial virus infection [1], influenza virus infection [2] and hepatitis C virus infection [3]. Recently, it has also been adopted to treat COVID–19 infected patients in combination with interferon beta–1b and lopinavir/ritonavir [4]. Due to the anti–viral properties, ribavirin has also been proven to be effective in treatment of avian influenza in poultry [5]. The accumulation of ribavirin residual in poultry meat could affect human health through the food chain, leading to hemolytic anemia [6], as well as cardiac and reproductive toxicity [7, 8]. Despite regulations and laws have been made in China and USA to prohibit the abuse of ribavirin, the illegal application of this drug cannot be completely eliminated. In China, it was reported by media that in order to make chicken grow faster and healthier, ribavirin, amantadine and antibiotics were added in chickenfeed, which is a violation of the Announcement No.560 issued by Ministry of Agriculture and Rural Affairs of the People’s Republic of China [9]. Consequently, it is of great importance to develop a qualitative and quantitative method for determination of ribavirin in poultry meat such as chicken.

To date, some methods have been developed to detect ribavirin in human biological samples and animal tissues [10]. Traditional methods, including UV detection [11], enzyme–linked immunosorbent assay (ELISA) [12], are two kinds of mostly used methods. Problematically, the above approaches may encounter some obstacles during detection. The drawback of UV–detection might be incomplete information about adsorption of compounds on the filter plates, which might lead to systematic error. ELISA may suffer from false positives due to protein cross–reactivity. Alternatively, DNA–based method, biosensors and liquid chromatography–mass spectrometry (LC–MS) have come into the front line of antiviral drug analysis during recent years [13–21], giving enhanced accuracy and better separation. Nevertheless, DNA–based methods can’t fully rule out contamination and false positive results, while biosensors are less competitive in multi–residue detection and sometimes are not suitable for high–throughput analysis. With regard to liquid chromatography–mass spectrometry (LC–MS) methods, the pre–treatment of samples and purification procedures can be very complicated and time–consuming. Another obstacle of LC–MS based methods is the matrix effect [22, 23], it is still challenging to separate ribavirin from biological samples because the internal interference (uridine) is similar in structure and will strongly affect mass spectrometric behavior of ribavirin, making both separation and quantification very tricky [24]. Despite the shortcomings of LC–MS technology, given properly developed pretreatment and purification procedures, it is delivering a superior advantage in anti–viral drug analysis with both accuracy and sensitivity. Meanwhile, mass spectrometers can monitor multiple transitions at one experiment, rendering high throughput drug screening possible.

In this study, a novel method for separation ribavirin in chicken has been developed and validated. The chicken samples were firstly extracted in 5% acetonitrile water with 0.1% formic acid, followed by purification with solid phase extraction (SPE) procedure using Hypercarb cartridge. The eluents were nitrogen dried and reconstituted in 1 mL 5mM ammonium acetate containing 5% acetonitrile (v/v) and 0.1%(v/v) formic acid. Chromatography separation was achieved on a Hypercarb column operated with acetonitrile (ACN)–ammonium acetate (5mM, with 0.1%(v/v) formic acid) mobile phase. The established method was fully validated by parameters such as LOQs, LODs, recovery rate and stability. Moreover, different brands of mass spectrometer were also compared with regarding to ribavirin separation, and both AB Sciex 5500 and Agilent 6495B have been proved to be suitable for separation ribavirin from chicken.

## Materials and methods

### Chemicals and reagents

Ribavirin and was obtained from Manhage Bio–Technology Co., LTD. (Beijing, China), The internal standard ^13^C_5_–ribavirin was purchased from TRC (TRC, Canada), methanol and acetonitrile (HPLC grade) were obtained from Honeywell (Honeywell, USA), formic acid (HPLC grade) was obtained from ACS (ACS, USA), ammonium acetate (≥ 99.0%, chromatographic grade) was purchased from Aladdin Chemical Reagent Co., Ltd. (Shanghai, China). Uridine (≥ 99.0%, Merck) was kindly supplied by Mr. Gordon Gao from TOFWERK (China). Ultra–pure water (18.2 MΩ·cm, TOC 1 − 2 ppb) was obtained from Milli–Q IQ7000 (Millipore, France).

### Spiked chicken samples

Negative chicken meat was obtained from local supermarket. Visible fat was trimmed off prior to homogenization using a laboratory blender (HM100, Grinder Instruments, Beijing, China). The spiked positive samples were obtained by adding intermediate ribavirin standard solution into the negative chicken samples prior to extraction. Before extraction by various solutions, 100 μL ^13^C_5_–ribavirin (1 μg/mL) was added to each weighed sample.

### Standard solutions

Stock solutions of ribavirin and internal standard (ISTD) ^13^C_5_–ribavirin were prepared in methanol at a concentration of 1000 μg/mL and stored at − 20 °C, respectively. Then, the intermediate standards solution of 1 μg/mL were obtained by diluting individual stock solutions with methanol, which were prepared immediately before use. Finally, the working solution of ribavirin was prepared by serial dilution of the ribavirin intermediate solution (1 μg/mL) with 5% acetonitrile water with 0.1% formic acid, and 50 μL of the intermediate ISTD solution (1 μg/mL) was added to each tube to obtain a concentration of 50.0 ng/mL.

### HPLC parameters

Three columns were used for method screening in the aim to separate ribavirin, namely Agilent ZORBAX SB–Aq (4.6 mm×100 mm, 1.8 μm, Agilent, USA), Waters BEH amide (2.1 mm×50 mm, 1.7 μm, Waters, USA), and Thermo Hypercarb column (2.1 × 100 mm, 5 μm, Thermo Scientific, USA). HPLC separations were performed on an above columns maintained at 40 °C using acetonitrile (ACN, phase A) and 5mM ammonium acetate containing 0.1%(v/v) formic acid (ammonium acetate, phase B) at a flow rate of 0.6 mL/min. Mobile phase gradient was: 5% A at 0 min, held at 5% A for 1 min, linear gradient to 70% A at 3.5 min, held at 70% A for 2.5 min, then returned to initial gradient 5% until 6 min. The injection volume was 5 μL.

### LC–MS/MS parameters

Two platforms of mass spectrometers were employed to evaluate the separation of ribavirin.

For Agilent mass spectrometer, samples were analyzed on an Agilent 1290 Infinity II UHPLC interfaced to an Agilent 6495 triple quadrupole mass spectrometer with Agilent’s Jet Stream source and operated in positive ionization (ESI+) mode (Agilent Technologies, Singapore). The parameters were as following: capillary voltage was 3000 V, nozzle voltage was 500 V, nebulizer pressure was 25 psi, sheath gas flow rate and temperature were 12 L/min and 350 °C, respectively. Dry gas flow rate and dry gas temperature were 130 °C and 17 L/min, respectively. Agilent Mass Hunter Softwares (LC/MS Data Acquisition, Version B.07.01; Quantitative Analysis, Version B.07.00) were employed to process mass data.

For AB SCIEX mass spectrometer, samples were loaded on a Exion LC interfaced to AB Sciex Triple Quad 5500 equipped with a Turbo V Spray source (AB Sciex, Singapore). Detection was achieved by multiple reaction monitoring (MRM) in positive ion mode with the following parameters: turbo gas temperature (TEM) was 500 °C, curtain gas (CUR) value was 35 psi, collision gas (CAD) was 9, ion spray voltage (IS) was 5000 V, declustering potential (DP) was 62 V. The Analyst software (Version 1.6.3) was used for data acquisition and Sciex OS software (Version 1.4.0) was used to process the acquired data.

Data was acquired under multiple reaction monitoring (MRM) mode, and the optimized ion parameters of ribavirin on both spectrometer platforms were shown in Table 1.

**Table 1:** MS parameters in multiple reaction monitoring (MRM) mode for ribavirin

Compound	Precursor ion (*m/z*)	Product ion (*m/z*)	Declustering Potential (V)	Collision energy (V)	Fragmentor* (V)
ribavirin	245.2	113**	62	13 (8*)	380
ribavirin	245.2	96.1	62	41 (44*)	380
^13^C_5_-ribavirin	250.13	112.9	62	16 (16*)	380

*Parameters on Agilent mass spectrometer, **Quantitative ion

### Sample preparation

2.00 g of homogenized chicken samples were weighed into a 50 mL polypropylene centrifuge tube, and 100 μL of ISTD (1 μg/mL) was added.

For hypercarb column purification method, extraction was initiated by adding 10 mL acidified Milli–Q water containing 0.1% formic acid and 5% acetonitrile. After vortex mixing for 1 min, the samples were centrifuged at 6000 rpm for 5 min. Afterwards, 5 mL of the supernatant was transferred into a 15 mL polypropylene centrifuge tube for further purification.

Before the clean–up procedure, the hypercarb cartridges (Hypercarb Hypersep, 200 mg, 3 mL, Thermo Scientific, USA) were pre–conditioned with 1 mL of methanol and 1 mL of acetonitrile. Then the 5mL supernatant was loaded on the column, the column was rinsed with 1 mL of methanol and 1 mL of acetonitrile before eluting the analytes with 1 mL of 20% acetonitrile water (2:8, v/v) containing 0.1% formic acid (v/v). The eluent was filtered through a 0.22–μm filter prior to LC–MS/MS analysis.

For PRiME HLB column purification method, extraction was started by adding 10 mL 80% acetonitrile water (8:2, v/v) containing 0.2% formic acid (v/v). After vortex mixing for 1 min, the samples were centrifuged at 6000 rpm for 5 min and 5 mL of the supernatant was passed through the Oasis PRiME HLB cartridges (6 mL, 500 mg). The eluents were collected immediately, nitrogen dried at 40 °C and reconstituted in 1 mL 5mM ammonium acetate containing 5% acetonitrile (v/v) and 0.1%(v/v) formic acid prior to LC–MS/MS analysis.

For phenyl boronic acid (PBA) column purification method, extraction was started by adding 10 mL 0.25mM ammonium acetate (pH 8.5) containing 0.1% formic acid. The samples were vortexed for 1 min and centrifuged for 5 min at 6000 rpm. Then 5 mL of the supernatant was loaded on the PBA cartridges (3 mL, 200 mg) sequentially pre–conditioned with 3mL acetonitrile, 3 mL acetonitrile: 1% formic acid (3:1, v/v) and 3 mL 0.25mM ammonium acetate (pH 8.5). After sample loading, the columns were washed with 3 mL of 0.25 mM ammonium acetate (pH 8.5) containing 10% acetonitrile, and 2 mL methanol containing 5% ammonia. The analytes were eluted into a 15 mL polypropylene centrifuge tube with 5.0 mL elution solution (formic acid, water, methanol; 2:8:90, v/v/v). Samples were dried under nitrogen air at 40 °C and reconstituted in 1 mL 5mM ammonium acetate containing 5% acetonitrile (v/v) and 0.1%(v/v) formic acid before LC–MS/MS analysis.

### Matrix effect

To evaluate the matrix effect, 2.00 g of homogenized chicken samples were weighed into a 50 mL polypropylene centrifuge tube followed by adding 10 mL acidified Milli–Q water containing 0.1% formic acid and 5% acetonitrile. After vortex mixing for 1 min, the samples were centrifuged at 6000 rpm for 5 min and the supernatant was taken as matrix. Matrix–containing calibration curves at seven concentration levels (1.0 ng/mL, 2.0 ng/mL, 5.0 ng/mL, 10.0 ng/mL, 20.0 ng/mL, 50.0 ng/mL, and 100.0 ng/mL) were analyzed. The matrix effect was obtained by comparing the slopes between the calibration curve in the matrix and in the solvent.

### Method validation

#### Linearity

In order to evaluate linearity of the method, matrix–matched (1:10 diluted extract) and external (solvent) standard calibration curves were assessed at seven concentration levels ranging from 1.0 ng/mL to 100.0 ng/mL. A regression plot was generated using peak area ratio versus ribavirin concentration and the regression coefficients (R^2^) was calculated.

#### Accuracy and precision

Accuracy and precision of the current established method were validated as per the FDA guidelines on bioanalytical method validation [25]. Intra and interday precision and accuracy were evaluated by injecting six replicates of QC samples spiked at four concentrations (LLOQ QC, 0.2 μg/kg; LQC, 0.5 μg/kg; MQC,15 μg/kg; HQC,200 μg/kg) on the same day and over three consecutive days. Accuracy and precision were expressed as the percentage error (RE %) and percentage relative standard deviation (RSD %), respectively.

#### Limits of detection (LOD) and quantitation (LOQ)

Limit of detection and quantification were estimated According to International Conference on Harmonization (ICH) guidelines. Briefly, the slope of the curve and the standard deviation of the intercept were used to calculate the LOD and LOQ based on the following equations: LOD = 3.3σ/s and LOQ = 10σ/s, where σ is standard deviation of intercept and s is the slope.

#### Stability

Freeze/thaw stability, room temperature stability, and extracted sample stability were evaluated using low and high QC samples [25]. Freeze and thaw stability was determined by freezing and thawing the low and high QC samples for three cycles before analysis at a 24 h interval. The initial concentrations of QC low/high samples were 1.00 ng/mL and 50.00 ng/mL, respectively. The QC low/high sample was prepared by serial dilution of 10 μL and 50 μL ribavirin intermediate solution (100 ng/mL and 1 μg/mL) with 5mM ammonium acetate containing 5% acetonitrile (v/v) and 0.1% (v/v) formic acid in 1 mL total volume. The concentration of internal ^13^C_5_–ribavirin was 50.0 ng/mL. To evaluate room temperature stability, low and high QC samples were exposed to ambient conditions for 120 h prior to extraction in triplicate. The mean response was compared with low and high QC samples that did not undergo treatment. Extracted sample stability was assessed by injecting of extracted QC samples after one–week storage in 20 °C. Long–term storage stability of ribavirin was determined by analyzing the QC samples on the day of preparation and after being stored at − 20 °C for 6 months.

## Results and discussion

### Optimization of LC–MS/MS conditions

In consistent with previous studies [26, 27], electrospray ionization (ESI) operating in positive mode is suitable for detection of ribavirin as it contains amino group, and gives (M + H)^+^ ions easily. The standard solution containing ribavirin and its isotopic internal standard ^13^C_5_–ribavirin was infused directly into mass spectrometer to optimize the parameters based on response with the optimization module of analytic software, the transitions of ribavirin and ^13^C_5_–ribavirin and MS parameters, were given in Table 1.

**Fig. 1:**
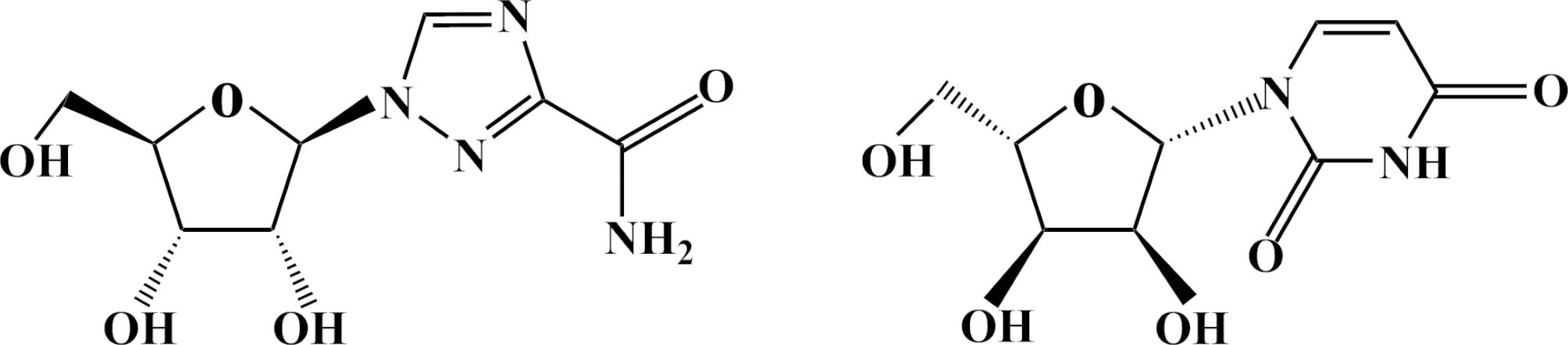
Chemical structures of ribavirin (left) and its analogue uridine (right)

Due to the strong polarity, ribavirin is water–soluble and can be easily interfered by endogenous impurities (uridine, Fig. 1; Supplementary Fig. 1). To elute ribavirin efficiently, combinations of different mobile phases, additives and gradients were compared. For aqueous solvents evaluation, water, water containing 0.1% (v/v) formic acid and 5 mM ammonium acetate containing 0.1% (v/v) formic acid were investigated based on several chromatographic parameters: resolution, symmetry factor and peak width. It turned out that 5 mM ammonium acetate containing 0.1% (v/v) formic acid yielded the best chromatographic behavior. In terms of organic solvents, both ACN and MeOH were checked with or without 0.1% (v/v) formic acid. Results revealed that acetonitrile was a more appropriate organic solvent for mobile phase during ribavirin separation due to better separation and higher resolution. While methanol gave a broader peak width of ribavirin, which would be disadvantageous in the separation of ribavirin and the internal interference (Supplementary Fig. 2). No distinguishable enhancement of ionization was observed while formic acid was added into the organic phase. In agreement with our results, previous study found that the biggest S/N of ribavirin had been acquired when 2.0 mM ammonium acetate solution/ACN had been used as mobile phase [28]. In our study, the final mobile phase combination was determined as phase A: ammonium acetate containing 0.1% (v/v) formic acid and phase B: acetonitrile.

Next, gradient elution program, flow rate, and column temperature were optimized with the aim of separating ribavirin from the endogenous interference and shortening the separation time. First, 95% aqueous phase and 5% organic phase was held for 1 min, then the organic phase ratio was increased to 70% during the next 2.5 min to ensure the separation of ribavirin from co–eluting interference (uridine). As can be seen from the total ion current (TIC) chromatogram in Supplementary Fig. 1A, the retention time of ribavirin and uridine were 2.38 and 2.75 min, respectively. This period was held for another 1.5 min, followed by a decrease to the initial ratio (95% ammonium acetate containing 0.1% (v/v) formic acid and 5% acetonitrile) in 1 min, giving a 6 min total run time. It was found that combinations of flow rate (0.6 mL/min), column temperature (40 °C) yielded a better separation and peak shape of ribavirin and uridine. Similar with our elution program, mobile phase gradient conditions in another study started with low organic phase B (A: 20 mM ammonium formate in water; B: acetonitrile) in the first minute and raised to 30% in the next 2.7 min, ramped up to 95% between 3.7 and 4.0 min and was held for 0.8 min, ramp down to 2% acetonitrile between 4.8 and 4.9 min, re–equilibrate at 2% acetonitrile from 4.9 to 5.5 min [13].

**Fig. 2:**
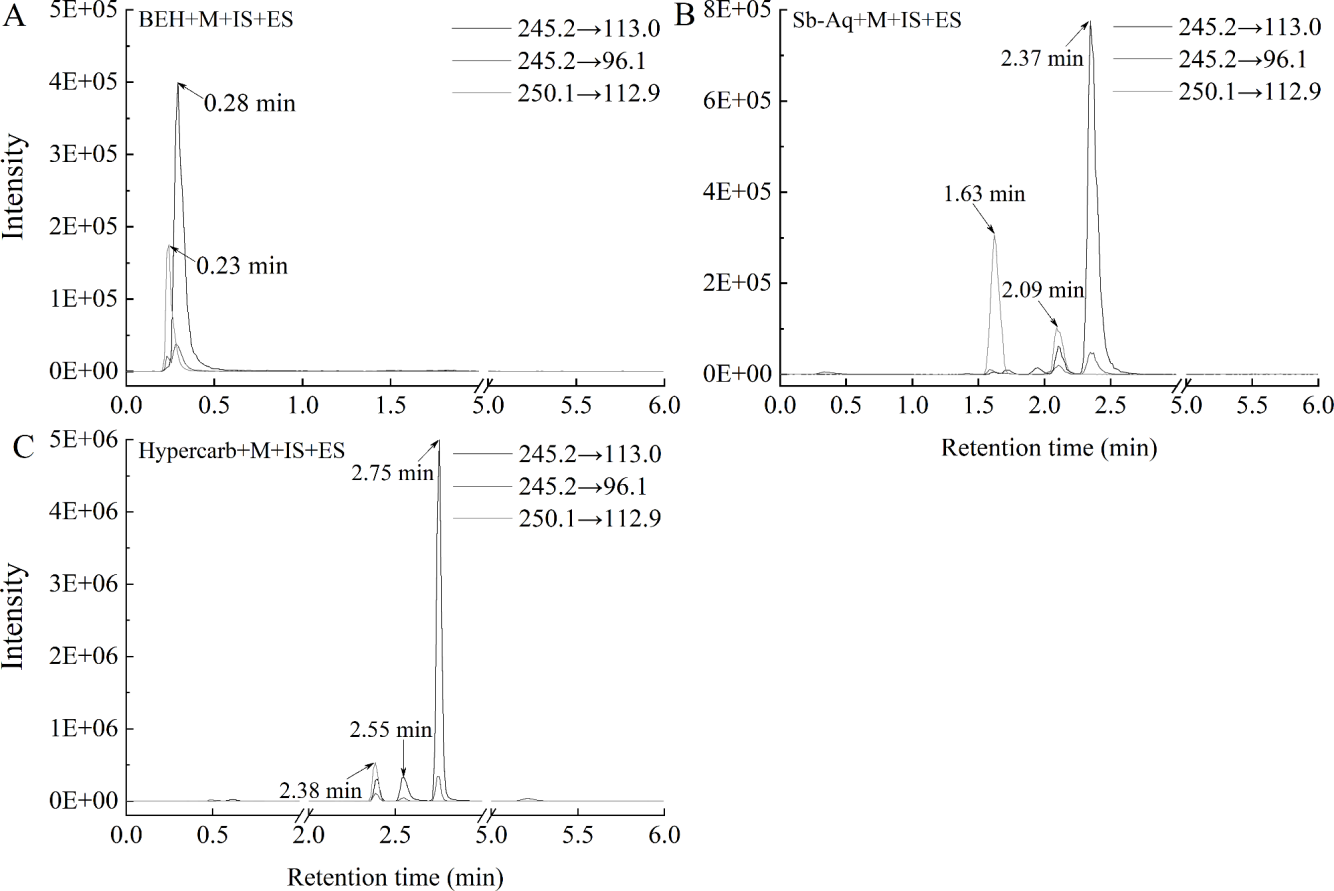
Effect of different analytical columns on the separation of ribavirin in spiked chicken samples. **A**: Waters BEH amide column (2.1 mm×50 mm, 1.7 μm), **B**: Agilent ZORBAX SB–Aq column (4.6 mm×100 mm, 1.8 μm), **C**: Thermo Hypercarb column (2.1 mm×100 mm, 5 μm). Blank chicken matrices (M) were spiked with ribavirin at the level of 5 μg/kg (ES) with 50.0 ng/mL of internal standard (ISTD) ^13^C_5_–ribavirin (IS). // indicated break of the time axis

Provided with the strong polarity of ribavirin, to obtain desirable separation and selectivity, different analytical columns were also evaluated. The Waters BEH (ethylene bridge hybrid) amide column (2.1 mm×50 mm, 1.7 μm), Agilent ZORBAX SB–Aq column (4.6 mm×100 mm, 1.8 μm), and Thermo Hypercarb (2.1 mm×100 mm, 5 μm) column were recruited to investigate the selectivity of ribavirin in chicken samples. BEH amide column contains trifunctionally bonded amide phase and is suitable for retention for polar analytes [29, 30]. SB–Aq column is a kind of alkyl reversed–phase bonded column designed to retain hydrophilic compounds when using aqueous mobile phases [31, 32]. Hypercarb column contains porous graphitic carbon sorbents and is believed to have better separation on polar compounds [33, 34]. As shown in Fig. 2A, it was found that at the spiking level of 5 μg/kg, BEH column failed to provide effective retention of ribavirin, not to mention the separation of target compound and its analogue interference. The two analytes almost merged into one peak and were co–eluted from the column at 0.29 min (Supplementary Fig. 3). The retention time for ribavirin standard was 2.09 min when it came to the SB–Aq column. In comparison with BEH column, it could distinguish ribavirin (2.10 min) from its internal interference uridine (2.36 min). However, there was an obvious interference peak (1.62 min) in the MRM channel of the ISTD (250.3→112.9, Fig. 2B, Supplementary Fig. 4). The adoption of Hypercarb column finally solved this problem, there is an obvious time gap between the two analytes, first was ribavirin at 2.39 min and then the uridine at 2.75 min. Importantly, no interfering peak was observed in the ISTD channel (Fig. 2C, Supplementary Fig. 5). In accordance with our results, it was reported that chicken muscle samples were extracted with methanol containing 1% acetic acid (v/v) and purified by a QuEChERS method using PSA and C18, before the separation was achieved on a Hypercarb analytical column under a gradient elution [35]. The method developed in our study was simple and comparable to the above QuEChERS method. More recently, another strategy, including extraction method followed by LC–MS/MS, was developed for the detection of five antiviral drugs in honey. After extraction with 1% formic acid and PBA cartridge purification, the target drugs were analyzed on an Agilent Poroshell 120 SB–Aq column (2.1 × 100 mm, 2.7 μm), a C18 surface–modified phase column, using 0.1% formic acid water (phase A) and methanol (phase B) [28]. The discrepancies could due to the different matrix and column properties as well as mobile phases and elution programs. Additionally, the Hypercarb analytical column, 20 mM ammonium formate as mobile phase A and 100% acetonitrile as moble phase B, togerther with similar elution gradients were also employed to separate ribavirin from the endogenous isobaric compounds in both human and bovine serum [13], indicating the broad matrix applicability of this method. Taken together, the Hypercarb column was chosen to analyze ribavirin in chicken samples.

### Optimization of sample preparation

To obtain desirable separation effect, the sample preparation step must also take into consideration. In vivo, ribavirin undergoes intracellular phosphorylation to mono–, di– and triphosphate moieties (RMP, RDP and RTP) respectively [36]. Therefore, it is necessary to dephosphorylate these metabolites into parent nucleoside moiety before separation and quantification. However, previous study proved that phosphorylation is not the pathway of ribavirin metabolism in chicken, as there was no significant difference between incurred chicken samples and samples without enzymatic hydrolysis [35]. In addition, in the present study, blank chicken samples spiked with ribavirin were used instead of incurred samples, so the determination of ribavirin does not require an enzymatic step using phosphoesterase.

Afterwards, the purification step was investigated by comparing different kinds of solid phase extraction (SPE) cartridges, with one key criteria: recovery rate of ribavirin. Namely, Hypercarb cartridge, PRiME HLB cartridge and PBA cartridge were evaluated with correspondent purification procedures.

Based on above SPE columns, three different extraction solvents were compared during sample preparation. Subsequently, extraction efficiency of water containing 0.1% formic acid and 5% acetonitrile, 80% acetonitrile water (8:2, v/v) containing 0.2% formic acid and 0.25mM ammonium acetate (pH 8.5) containing 0.1% formic acid were evaluated, respectively. Results revealed that high proportion of organic phase in extraction solution (80% acetonitrile water) plus PRiME HLB clean–up strategy yielded a recovery rate of only 25.46% (Table 2). PRiME HLB cartridge is not a conventional hydrophilic lipophilic balance (HLB) column. It can remove fat and phospholipids efficiently and doesn’t require conditioning or equilibration prior to sample loading. We wonder whether non–necessity in conditioning of PRiME HLB cartridge could have influence on the recovery rate of ribavirin. To rule out this possibility, PRiME HLB columns were preconditioned with 1mL methanol and 1 mL ultrapure water. Subsequent data indicated that there was no obvious difference between conditioned and un–conditioned PRiME HLB cartridge with regard to recovery rate (data not shown). Although PRiME HLB was able to extract up to sixteen cephalosporins in milk with relative recoveries ranging from 56 to 93% [37], it was not suitable in the scenario of ribavirin separation, probably due to the lack of interaction between packing materials and target compound. The recovery rate of extraction solution consisting of 0.25mM ammonium acetate (pH 8.5) containing 0.1% formic acid followed by PBA purification was 75.32%. PBA cartridge provides reversible covalent bonding with sufficient affinity for aromatic o–hydroxy compounds. Under alkaline conditions, the two hydroxyl groups on the immobilized phenylboronic acid molecule can bond to diols (such as ribavirin) covalently [38]. Uridine could also be retained on PBA column as it has a similar structure as ribavirin. Moreover, ribavirin base was expected to be present in the wash waste during the loading step in SPE due to its lack of poly–ol groups in the molecule and competitive retaining strength with other impurities such as glucose [28, 39]. In addition, PBA strategy, in which samples must be loaded with under alkaline condition and eluted under acidic condition, requires numerous traditional steps including conditioning, equilibrating, loading, washing, and eluting procedures. Alternatively, Hypercarb is a kind of cartridge containing porous graphitic carbon (PGC) sorbents. The PGC has increased retention for polar substances as it contains layers of hexagonally arranged carbon atoms which make the content of polar groups low, and consequently the solutes mainly interact with the PGC–surface by π–π electron interactions [40]. As can be seen from Table 2, water containing 0.1% formic acid and 5% acetonitrile as extraction solution and Hypercarb cartridge as clean–up measure showed the best peak shape and highest recovery rate (85.68%) of ribavirin at spiked level of 5 μg/kg. In comparison with PBA method, this strategy is not time–consuming as it doesn’t require washing and nitrogen drying process. Noteworthily, results showed that despite different extractants and clean–up steps, there is an obvious interference peak not far from peak of ribavirin, the interference peak was higher than the target peak. If this peak is taken into account, typically merged into one peak with target compound under unoptimized conditions, the accurate quantification of ribavirin is impossible and false positive results will be inevitable. Based on the above results, water containing 0.1% formic acid and 5% acetonitrile was chosen as the final solvent to extract ribavirin from chicken, and Hypercarb cartridge was selected for purification.

**Table 2:** Recovery rates of ribavirin from chicken samples spiked at 5 μg/kg level

Cartridge	Specifications	Recovery (%)	RSD (%)
PriME HLB	500 mg/6 mL	25.46	3.12
PBA	200 mg/3 mL	75.32	2.96
Hypercarb	200 mg/3 mL	85.68	4.05

### Method validation

To evaluate the applicability of the developed method, linearity, accuracy, precision, limit of detection (LOD), limit of quantification (LOQ), as well as matrix effect and stability were investigated.

#### Linearity

The non–matrix based and matrix–matched calibration curves of ribavirin were prepared over a linear range from 1.0 ng/mL to 100.0 ng/mL at seven concentrations with the concentration of internal standard ^13^C_5_–ribavirin at 50.0 ng/mL. A regression plot was generated using the ratio of the areas of the analyte and the internal standard peaks versus the concentration. The calibration curves showed good linearity with correlation coefficients (R^2^) greater than 0.999 in all the cases.

#### Accuracy and precision

The intraday precision and accuracy were confirmed using replicates of QC samples (n = 6) at four levels of concentrations (LLOQ QC, LQC, MQC and HQC). All replicates at these four concentration levels from three separate days were used to evaluate the interday precision. The results were in the acceptable range according to the FDA guidelines. The accuracy results were found to be − 7.83 − 1.39% with precision ranging from 1.34 − 3.88% during intraday analysis. The accuracy results were found to be − 6.38 − 2.25% with precision ranging from 1.10 − 4.67% during interday analysis (Table 3).

**Table 3:** Intraday and interday accuracy and precision of ribavirin from spiked chicken samples (mean ± SD, n = 6)

Analyte	QC	Spiking level (?g/kg)	Intraday (n = 6)			Interday (n = 6)		
			Found (μg/kg; mean ± SD)	Accuracy (RE %)	Precision (RSD %)	Found (μg/kg; mean ± SD)	Accuracy (RE %)	Precision (RSD %)
**Ribavirin**	LLOQ QC	0.2	0.1843 ± 0.0070	−7.83	3.80	0.1873 ± 0.0087	−6.38	4.67
	LQC	0.5	0.4657 ± 0.0181	−6.87	3.88	0.4719 ± 0.0207	−5.62	4.39
	MQC	15	14.40 ± 0.3372	−3.99	2.34	14.61 ± 0.4009	−2.58	2.74
	HQC	200	202.8 ± 2.7143	1.39	1.34	201.5 ± 3.2029	2.25	1.10

LLOQ QC, lower limit of quantitation quality control; LQC, low quality control; MQC, medium quality control; HQC, high quality

control. RE, relative error; RSD, relative standard deviation

#### LODs and LOQs

The limit of detection (LOD) was defined as the lowest concentration of ribavirin that generated a signal to noise (*S/N*) ratio greater than 3, while the limit of quantitation (LOQ) was considered as the lowest concentration of ribavirin that generated a signal to noise (*S/N*) ratio greater than 10. Result revealed that, the LOD and LOQ of ribavirin was 0.1 ng/mL and 0.5 ng/mL, respectively. Previous research reported that the CCα (defined as the limit above which it can be concluded with an error probability of α that a sample is non − compliant) and CCβ (defined as the value of CCα plus 1.64 times the standard deviation of the within–laboratory reproducibility of the ribavirin (5.0 μg/kg)) of a method designed for ribavirin detection in chicken muscle were 1.1 μg/kg and 1.5 μg/kg, respectively (Wu et al. 2016). In another work the LOD and LOQ of ribavirin in honey was 2 μg/kg and 5 μg/kg, respectively [28]. The LOD and LOQ in present study were superior in comparison with those previous results.

#### Matrix effect

Matrix effects (ME), represented by either enhancement or suppression of the ionization signals for the analytes in LC–MS/MS analysis, especially in ESI mode, is mainly caused by co–eluted substance in the matrix [41]. To evaluate the matrix, the slope of the matrix–matched calibration curves at seven concentration levels (1.0 ng/mL, 2.0 ng/mL, 5.0 ng/mL, 10.0 ng/mL, 20.0 ng/mL, 50.0 ng/mL, and 100.0 ng/mL) of ribavirin and its isotopic internal standard (50.0 ng/mL) was compared with those obtained in pure solvent, and matrix effect was evaluated according to previous report [42]. The results showed that the ME of ribavirin was 0.62, which suggested the existence of a significant matrix suppression effect. In line with our results, matrix suppression of ribavirin was also found in biological samples including honey [28], human serum [43].

#### Stability

Conditional stability of ribavirin was investigated by assessing room temperature stability, freeze/thaw stability and extracted sample stability. QC samples prepared in triplicate at both low and high concentrations were recruited to evaluate the stability. Room temperature stability was analyzed by maintaining samples at ambient condition (24 °C) for 120 h prior to extraction in triplicate. Freeze/thaw stability was determined by freezing and thawing the QC samples for three cycles before extraction in triplicate. Extracted sample stability was assessed by preserving three replicate extracted QC samples in the auto–sampler held at 20 °C for a week before reinjection. Mean response was compared to both nominal and mean response from freshly prepared QC samples at the same levels that were not subjected to the test condition. As shown in Table 4, ribavirin was considered to be stable under these conditions, as the deviation from control in all three experiments ranging from 0.13 to 6.33%.

**Table 4:** Stability of ribavirin under various conditions

**Condition**	**Found (ng/mL)**	RE (%)	CV (%)
	**QC low QC high**	**QC low QC high**	**QC low QC high**
**Freeze/thaw** **120 h RT** **One − week storage**	1.03 50.09 1.05 50.12 1.06 50.07	2.67 0.19 5.00 0.24 6.33 0.13	2.34 0.08 3.43 0.16 4.24 0.10

Freeze/thaw, 3 freeze (− 20 °C)/thaw cycles; one − week storage (20 °C). The initial concentrations of QC low/high samples were 1.00 ng/mL and 50.0 ng/mL, respectively. The concentration of internal standard (ISTD) ^13^C_5_–ribavirin was 50.0 ng/mL. RE, relative error; CV, coefficient of variation; RT, room temperature (20 °C)

Additionally, long term stability of ribavirin was also examined by analyzing the both QC samples (matrix) and ribavirin stock solutions (acetonitrile) on the first day of preparation and after being stored at − 20 °C for 6 months. Ribavirin was found to be stable in both matrix and pure solvent (Table 5).

**Table 5:** Long term stability of ribavirin in solvent and chicken matrix

**Matrix**	**Found (ng/mL)**	RE (%)	CV (%)
	QC low QC high	QC low QC high	QC low QC high
**Acetonitrile (0.5%, 0.1%FA)**	0.97 49.92	−3.33 −0.17	1.58 0.09
**Chicken** matrix	0.94 49.66	−6.33 −0.67	2.22 0.27

Long term, six − month storage (− 20 °C)

#### Application of the method

The established method has been applied to analyze ribavirin residue in chicken samples obtained from local market. A batch of 50 chicken samples were collected and subjected to analysis. Results showed that three chicken samples were tested positive for ribavirin, the concentrations of ribavirin were 2.6 μg/kg, 5.8 μg/kg and 0.9 μg/kg, respectively. These results suggested that ribavirin has been adopted in chicken breeding industry, and the positive rate was 6.0%.

### Comparison of different mass spectrometer platforms

After optimization of the LC–MS/MS condition and sample preparation, different brands of mass spectrometer platforms were also compared using the optimized method. The Agilent 6495B mass spectrometer and AB Sciex TripleQuad 5500 mass spectrometer were employed during comparison, on the basis that they have equivalent discrimination power. The LC parameters, such as gradient elution program, flow rate and column temperature were all set to the same values. Chicken samples were extracted and purified using the method described before, afterwards the samples were divided into two equal portions and loaded on the two mass spectrometers simultaneously. By setting the above parameters and procedures, many of the confounding factors that could occur when analyzing a single sample on different platforms were ruled out. Results shown that ribavirin and uridine could have good separations on both platforms. The linearity of calibration curves was good on both platforms, with the correlation coefficient (R^2^) greater than 0.99 (0.9993 and 0.9974). The recovery rates at spiking level of 5.0 μg/kg were 95.6% and 96.3%, respectively. Based on the above results, we recommend both of the above mass spectrometers as the appropriate platforms for analysis of ribavirin.

## Conclusions

In this work, a sensitive method using LC–MS/MS was developed for the detection of ribavirin in chicken samples. The sample extraction and purification procedures were simple and fast compared to the previous reported methods, and with tailored LC–MS/MS conditions, good separations of ribavirin and its internal interference was achieved. The current method provided high recovery, good stability, excellent accuracy and precision. The matrix effected was also carefully studied and the method was validated by analyzing chicken samples obtained from the local market. The application of this method was further addressed by comparing the performance of different mass spectrometers. We propose this method as a useful tool to monitor ribavirin in chicken samples.

### Electronic supplementary material

Below is the link to the electronic supplementary material.

Supplementary Material 1

## Data Availability

The datasets analyzed during the study are available from the corresponding author upon reasonable request.
