# Supplementary material for: LC–MS/MS separation and quantitation of ribavirin in chicken and comparison of different mass spectrometric platforms
Source: BMC Chem. 2023 Aug 7;17(1):96. doi: 10.1186/s13065-023-01010-4 (PMC10408068; doi:10.1186/s13065-023-01010-4)

Supplementary Figure 1. Total ion current (TIC) chromatogram of the chicken sample spiked with ribavirin and its ISTD ribavirin–^13^C_5_ (A); uridine with ISTD (B) and uridine only (C). Pure uridine (100 ng/mL) was prepared in 5mM ammonium acetate containing 5% acetonitrile (v/v) and 0.1%(v/v) formic acid and the concentration of ribavirin–^13^C_5_ was 50.0 ng/mL



Supplementary Figure 1.

Supplementary Figure 2. Total ion current (TIC) chromatogram of ribavirin separated using methanol (A) and acetonitrile (B) as organic phase. Ribavirin prepared in concentration of 10.0 ng/mL (with 50.0 ng/mL ISTD ribavirin–^13^C_5_) was injected into mass spectrometer using methanol or acetonitrile as organic phase.

Supplementary Figure 2.





Supplementary Figure 3. MRM chromatogram of ribavirin separated on Waters BEH amide analytical column (2.1 mm×50 mm, 1.7 μm). A: ribavirin standard solution (STD), B: blank chicken matrices, C: blank chicken matrices with ISTD. Blank chicken matrices (M) were spiked with 50.0 ng/mL of internal standard (ISTD) ribavirin–^13^C_5_ (IS). // indicated break of the time axis.


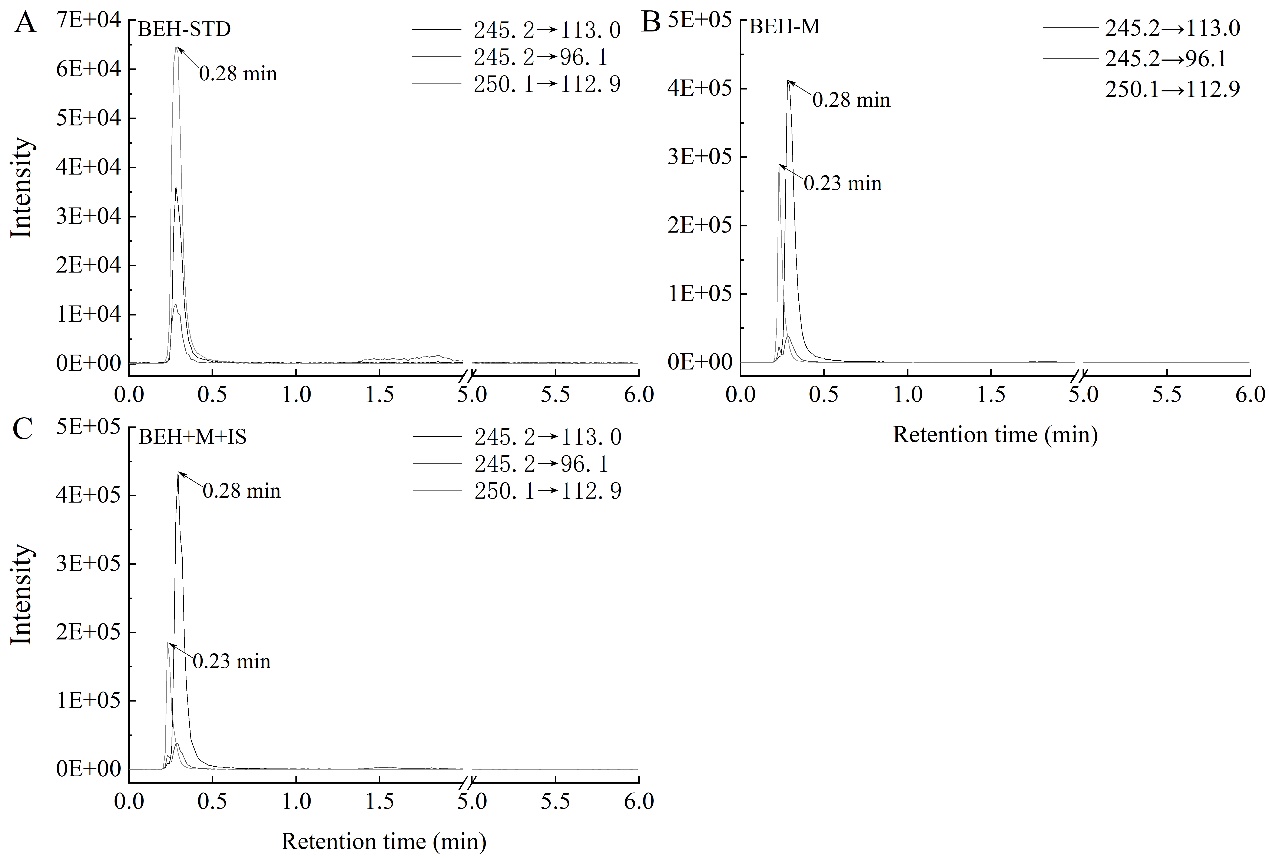
Supplementary Figure 3.

Supplementary Figure 4. MRM chromatogram of ribavirin separated on Agilent ZORBAX SB–Aq analytical column (2.1 mm×50 mm, 1.7 μm). A: ribavirin standard solution (STD), B: blank chicken matrices, C: blank chicken matrices with ISTD. Blank chicken matrices (M) were spiked with 50.0 ng/mL of internal standard (ISTD) ribavirin–^13^C_5_ (IS). // indicated break of the time axis.

Supplementary Figure 4.


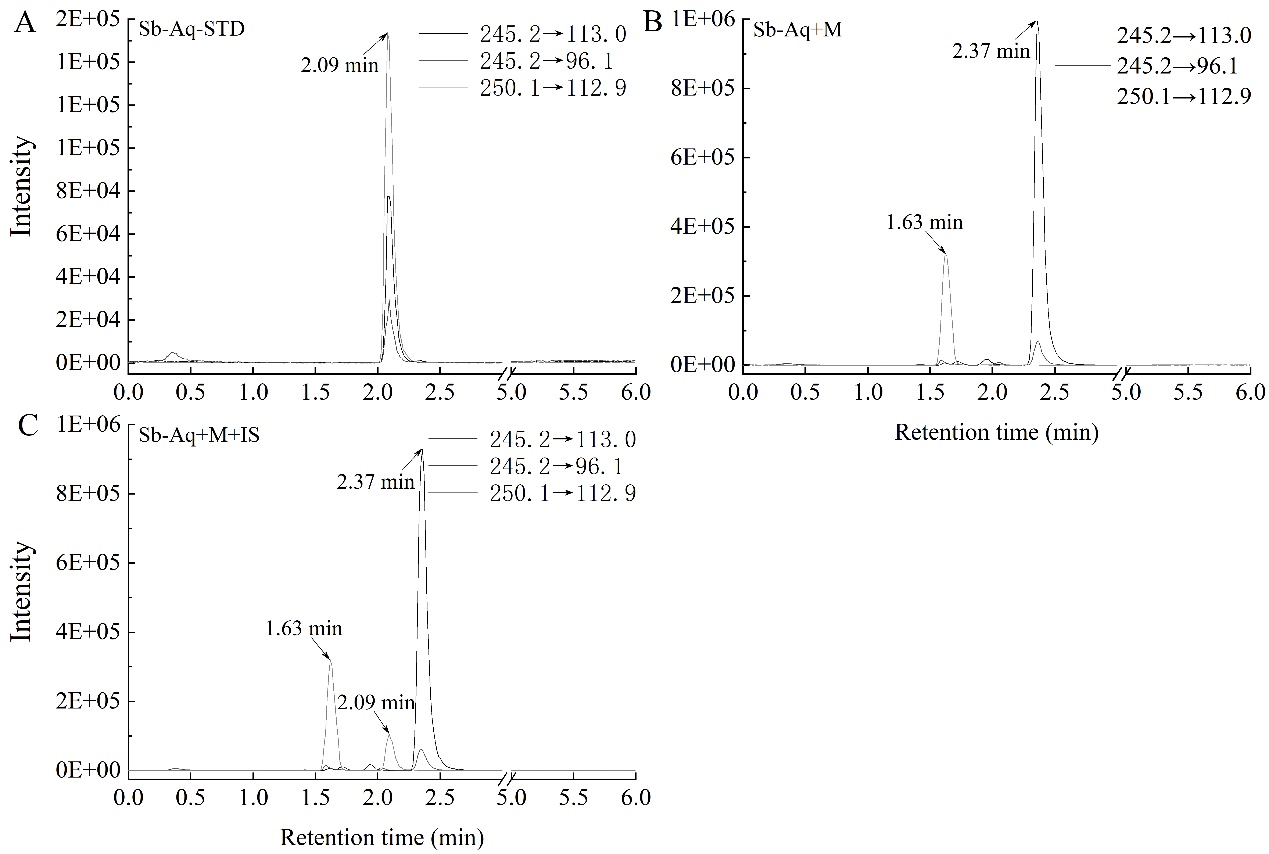


Supplementary Figure 5. MRM chromatogram of ribavirin separated on Thermo Hypercarb analytical column (2.1 mm×100 mm, 5 μm). A: ribavirin standard solution (STD), B: blank chicken matrices, C: blank chicken matrices with ISTD. Blank chicken matrices (M) were spiked with 50.0 ng/mL of internal standard (ISTD) ribavirin–^13^C_5_ (IS). // indicated break of the time axis.

Supplementary Figure 5.


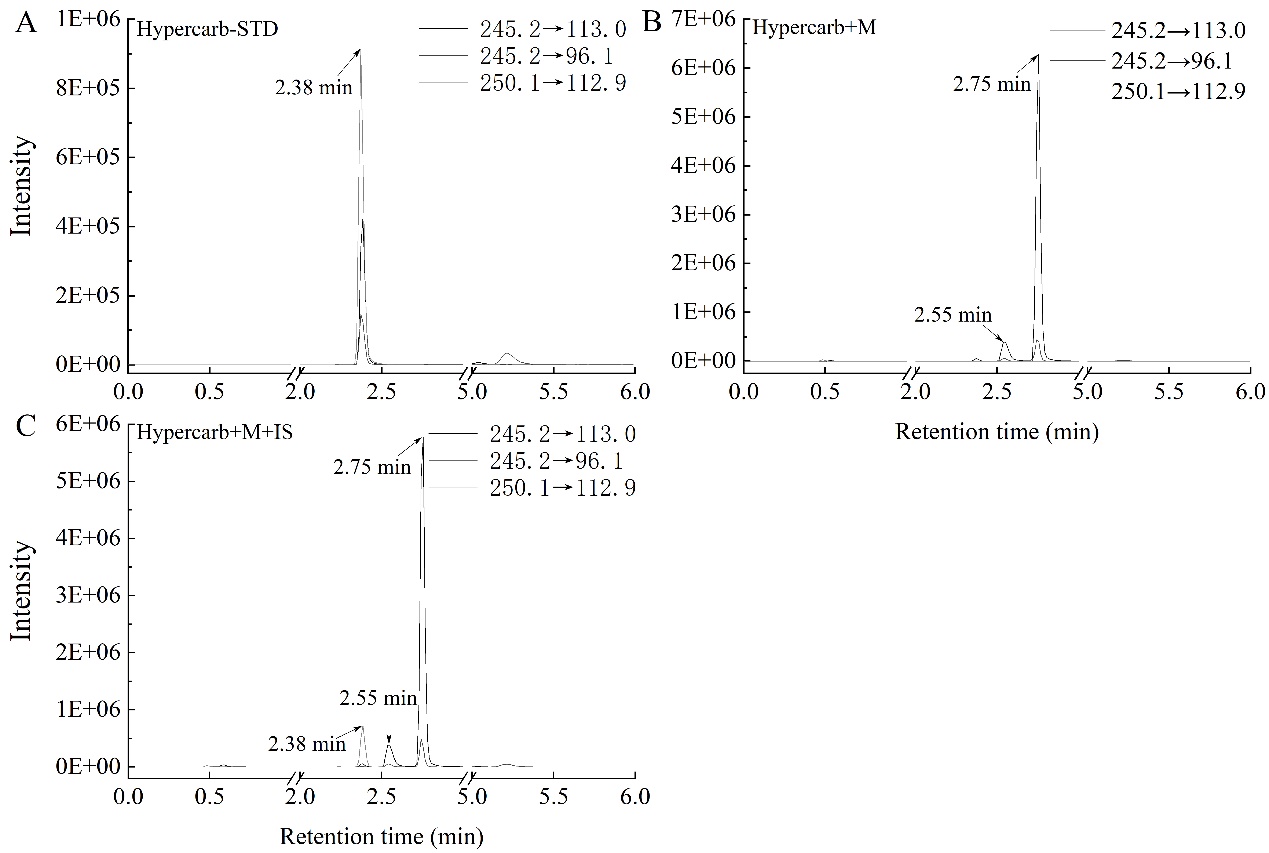

Supplement: Supplementary file 1 — Supplementary Material 1 [file 13065_2023_1010_MOESM1_ESM.docx]
